# Contemporary methods for ancient problems: AI for AKI

**DOI:** 10.1186/s40635-026-00858-9

**Published:** 2026-01-26

**Authors:** Georg Franz Lehner, Timo Mayerhöfer, Fabian Perschinka, Michael Joannidis

**Affiliations:** Division of Intensive Care and Emergency Medicine, Department of Internal Medicine, Medical University Innsbruck, 6020 Innsbruck, Austria

Early identification of acute kidney injury (AKI) remains one of the central challenges in critical care. The current definition of AKI is still based on creatinine and urinary output (UO) with their well-known limitations [1]. In their recent article, Koozi et al. tackle this highly relevant topic with artificial intelligence (AI) models [2].

They aimed to investigate predictors of new-onset AKI and the need for renal replacement therapy (RRT) using an explainable artificial intelligence (XAI) approach based on eXtreme Gradient Boosting (XGBoost). To uncover the most influential predictors, they employed SHapley Additive exPlanations (SHAP) and benchmarked their models against traditional logistic regression approaches. The authors included a comprehensive set of parameters comprising traditional diagnostic criteria for AKI, UO, and creatinine, but also more advanced renal biomarkers such as neutrophil gelatinase-associated lipocalin (NGAL) or cystatin C. Of note, creatinine did not turn out to be among the top predictors of new-onset AKI.

In the current study, the XGBoost models outperformed traditional logistic regression models with the same variables, both for new-onset AKI and renal replacement therapy (RRT). While the area under the curves (AUCs) for AKI prediction were similar to those reported in a recent systematic review by Cama-Olivares et al. [3], the AUCs for RRT prediction of the current study are particularly noteworthy.

AI methods may play an important role in detecting patients at risk for AKI earlier than clinical criteria alone [4]. The study by Koozi et al. also demonstrates a key strength of AI models in this context: They can decipher subtle changes, specific patterns, and particular combinations. Importantly, the models are able to reveal clinically relevant non-linear relationships.

Such interesting findings were observed in the case of endostatin, where the combination of high endostatin and low creatinine was associated with a higher risk for RRT than the combination of high endostatin and high creatinine.

The authors also report a particular lactate pattern, where lower levels of lactate, even within the normal range (up to 1.4 mmol/l), appear to have an attenuating predictive influence in their model. In other words, low lactate may diminish the predictive contribution of other variables. The analysis also revealed a remarkable non-linear relationship between AKI and UO with an inflexion point of 1500 mL urine per day, which might imply that a threshold above the Kidney Disease: Improving Global Outcomes (KDIGO) defined 0.5 ml/kg/h might more accurately indicate the risk for early AKI.

Several recent studies highlight the potential of advanced statistical and machine-learning techniques to unravel the biological heterogeneity of AKI beyond single-biomarker approaches [5]. Although promising, such advanced methods come with important limitations. Generalizability is often limited, particularly when models are trained on single-center or retrospective datasets and, thus, prone to bias. Also, the findings of the current study still require confirmation in an independent cohort. Implementing such complex approaches in clinical practice will also be challenging.

Nevertheless, the work by Koozi et al. illustrates how contemporary computational tools can tackle long-standing clinical problems and potentially advance risk stratification and early precise interventions in the future. If validated, such models could even support clinical decision-making regarding the initiation of RRT.

## Data Availability

Not applicable.
